# Management of hyperparathyroidism (PHP) in MEN2 syndromes in Europe

**DOI:** 10.1186/1756-6614-6-S1-S10

**Published:** 2013-03-14

**Authors:** Maria Alevizaki

**Affiliations:** 1 Endocrine Unit, Evgenidion Hospital and Dept Medical Therapeutics, Alexandra Hospital, Athens University School of Medicine, 11528 Athens, Greece

## Abstract

Hyperparathyroidism occurs in 20-30% of MEN2A syndrome patients. It is usually associated with mild disease and is frequently asymptomatic, especially in younger age. There is genotype/phenotype association and PHP is usually associated with codon 634 mutations; however association with more “rare” mutations has also been reported. The pathology of the parathyroid glands includes hyperplasia, adenoma or a combination of the two. The optimal surgical management of this entity has not been defined yet.

## Background

The parathyroids are involved in the multiple endocrine neoplasia (MEN2) syndromes more rarely than the other two endocrine organs affected (c cells and adrenal medulla). The prevalence of PHP varies between 20-30% in MEN2A. In the majority of cases hyperplasia precedes the development of adenoma formation. However both may co-exist or even a single adenoma may be found at operation. One of the clinical features characteristic of primary hyperparathyroidism in the context of the MEN2 syndromes is the younger age at presentation. European studies have shown that the majority of cases are diagnosed before age 39; furthermore, most of the patients are asymptomatic at PHP diagnosis [1]. A few studies have been published concerning associations between specific ret codons and the occurrence of PHP in familial MTC. It appears that there is a genotype/phenotype correlation concerning this manifestation of MEN2, as the majority of PHP cases cluster with exon 11 mutations and specifically with the 634 codon mutation [2-4]. Hyperparathyroidism has occasionally been reported in association with other ret gene mutated codons such as codons 611, 618, 620, 630 etc.

One of the important issues concerning the management of PHP in MEN syndromes in general is the ability to recognize single gland versus multiple gland disease and subsequently decide on the surgical procedure to be followed so that recurrences can be avoided. The following procedures are applied in practice depending on the number of glands involved: total parathyroidectomy with autotransplantation, subtotal parathyroidectomy, or single gland removal. One other problem is the occurrence of permanent hypoparathyroidism in some cases; this complication is usually associated with total parathyroidectomy. However, hypoparathyroidism has also been reported after subtotal parathyroidectomy, or even after excision of 2 glands [5]. Re operation can be performed when recurrent disease is diagnosed, but this is not always followed by cure. Successful use of calcimimetics has been reported in cases of disease persistence [6].

The use of total parathyroidectomy with autotransplantation at the time of primary surgery is popular in some centres [5,7], but is not routine practice in Europe [3,8,9].

### Comments to the ATA guidelines

There are no major differences between the European approach and the ATA approach on this issue. It is interesting that all the recommendations are grade C. Recommendation 26: screening for PHP is necessary in asymptomatic *ret* mutation carriers. The ATA proposes to start the screening by age 8 in the cases carrying mutations in those codons which are frequently associated with the occurrence of PHP.

Comment: universally starting at the age of 8 years appears a little early as the median age at presentation is 38 years [10]. There are only few cases of PHP presenting before the third decade; furthermore PHP appears to be generally mild in young subjects with MEN2 syndrome [1]. We thus believe age 20 seems more appropriate if there are no specific family data for earlier appearance. Yearly checking is optimal as the calcium estimation is an inexpensive and simple blood test.

Recommendations 47-50. These recommendations concern the optimal surgical treatment for PHP in MEN2.

Comment. The optimal surgical treatment is not yet defined especially in a previously thyroidectomised patient; therefore, the consequences of the PHP in these patients should be assessed per individual patient and the decision for surgery should be reached accordingly. This view is based on the fact that there is a substantial rate of both permanent hypoparathyroidism as well as recurrent/persistent hyperparathyroidism in most published series [3,5]. On the other hand in the majority of young patients the hyperparathyroidism is “asymptomatic” [10]. In recent years less invasive methods are used [9].

## List of abbreviations used

MEN2: multiple endocrine neoplasia type 2; MTC: medullary thyroid carcinoma; PHP: primary hyperparathyroidism.

## Competing interests

No competing interest are declared by the author of this paper.

## Declarations

This article has been published as part of *Thyroid Research* Volume 6 Supplement 1, 2013: European comments on Medullary Thyroid Cancer Management Guidelines of the American Thyroid Association. The full contents of the supplement are available online at http://www.thyroidresearchjournal.com/supplements/6/S1. Publication of this supplement has been supported by the European Thyroid Association-Cancer Research Network.
